# A Parallel

**Published:** 1886-01

**Authors:** 


### A PARALLEL.

Opium, or some of its derivatives, is almost universally acknowledged to be *the antagonist of pain*, in its manifold expressions; known to possess the power of *relieving pain* in the most pronounced manner; and to more profoundly impress the nervous system, whose various pathological conditions are expressed by pain, than perhaps any or all other therapeutic agencies known to man. So well recognized is this fact, that not to make use of it in a neuralgia of a vital organ seriously affecting life, but depending on, in its stead, infinitesimal portions of drugs which are supposed to have *opposite* effects;—that is to say, drugs possessing, according to the homœopathic dogma, the power of *producing pain* in a part, if taken in health, corresponding to the part affected, and for which it is prescribed, is, in our judgment, a very near approach to *criminality*. It can well be compared to the use of ten drop spirts of turpentine, benzine, coal oil, or *whisky*, thrown on a building in flames, to the neglect of the hydrant and hose which stand available at every corner. What would be the judgment of a court, for illustration, if an insurance company refused payment of a policy, setting up in justification, that the owner, instead of resorting to a sufficient quantity of water, which was readily available, had simply thrown a few teaspoonfuls of inflamable liquid on his building, under the pretext that he was following a rational course, with a view to extinguishing the flame? We submit that the parallel is exact, and represents the absurdity of giving simples, "which had no more effect than the remedies suggested by the women of the family," in a case of neuralgia of the heart, such as destroyed Gen. McClelland's life, while the homœopath stood by "powerless to relieve him."

The above is suggested by the criticisms (newspaper) of our leader in last month's issue, wherein we say that "a hypodermic of morphia and atropia, at once a powerful heart stimulant *and sedative*, would, *in all probability*, have saved that valuable life."

